# Bioactivity-Guided Isolation of Totarane-Derived Diterpenes from *Podocarpus neriifolius* and Structure Revision of 3-Deoxy-2*α*-hydroxynagilactone E

**DOI:** 10.1007/s13659-019-0198-x

**Published:** 2019-02-19

**Authors:** P. Annécie Benatrehina, Wei-Lun Chen, Austin A. Czarnecki, Steven Kurina, Hee-Byung Chai, Daniel D. Lantvit, Tran N. Ninh, Xiaoli Zhang, Djaja D. Soejarto, Joanna E. Burdette, A. Douglas Kinghorn, L. Harinantenaina Rakotondraibe

**Affiliations:** 1 Division of Medicinal Chemistry and Pharmacognosy, The Ohio State University, College of Pharmacy, Columbus, OH USA; 2 Center for Biostatistics, The Ohio State University, Columbus, OH USA; 3 College of Pharmacy, University of Illinois at Chicago, Chicago, IL USA; 4 John G. Searle Herbarium of the Field Museum of Natural History, Chicago, IL USA; 5 Institute of Ecology and Biological Resources, Vietnam Academy of Science and Technology Hanoi, Hanoi, Vietnam

**Keywords:** *Podocarpus neriifolius*, Nagilactone G-2*β*-*O*-*β*-d-glucoside, Hollow fiber assay, 3-Deoxy-2*α*-hydroxynagilactone E, Antiproliferative, B-type podolactone

## Abstract

**Abstract:**

Bioactivity-guided phytochemical investigation of *Podocarpus neriifolius* D. Don. (Podocarpaceae) has led to the isolation of one new (**2**) and three known (**1, 3,** and **4**) B-type podolactones, along with three totarane-type diterpenes (**5-7**). Their structures were determined by interpretation of High Resolution ElectroSpray Ionization Mass Spectrometry (HRESIMS) and 1D and 2D NMR data, and comparison with the values reported in the literature. The structure of compound **1**, previously identified as 3-deoxy-2*α*-hydroxynagilactone E (**8**), was revised as its 2*β*-epimer, which has been reported recently as a new compound. All of the isolates were evaluated for their antiproliferative activity against a panel of four human cancer cell lines, namely, ovarian (OVCAR3), breast (MDA-MB-231), colon (HT-29), and melanoma (MDA-MB-435), and compounds **1** and **3** were found to be cytotoxic with IC_50_ values in the low micromolar range for most of the cell lines used. The major compound, inumakilactone A (**3**), was further tested in vivo using the HT-29, MDA-MB-435, and OVCAR3 cells in a murine hollow fiber model, for the first time.

**Graphical Abstract:**

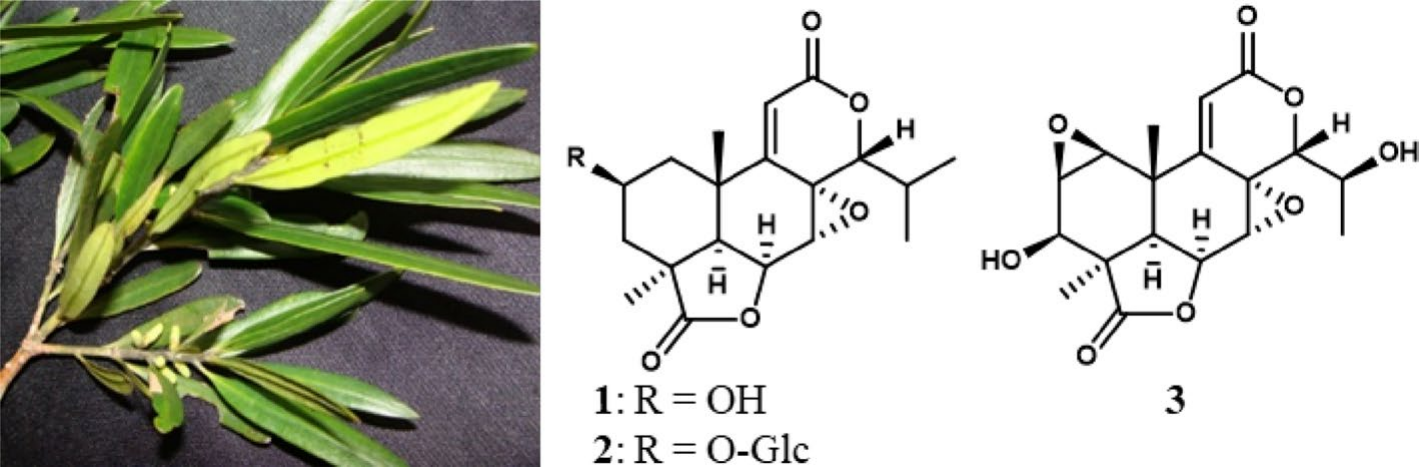

**Electronic supplementary material:**

The online version of this article (10.1007/s13659-019-0198-x) contains supplementary material, which is available to authorized users.

## Introduction

**Fig. 1:**
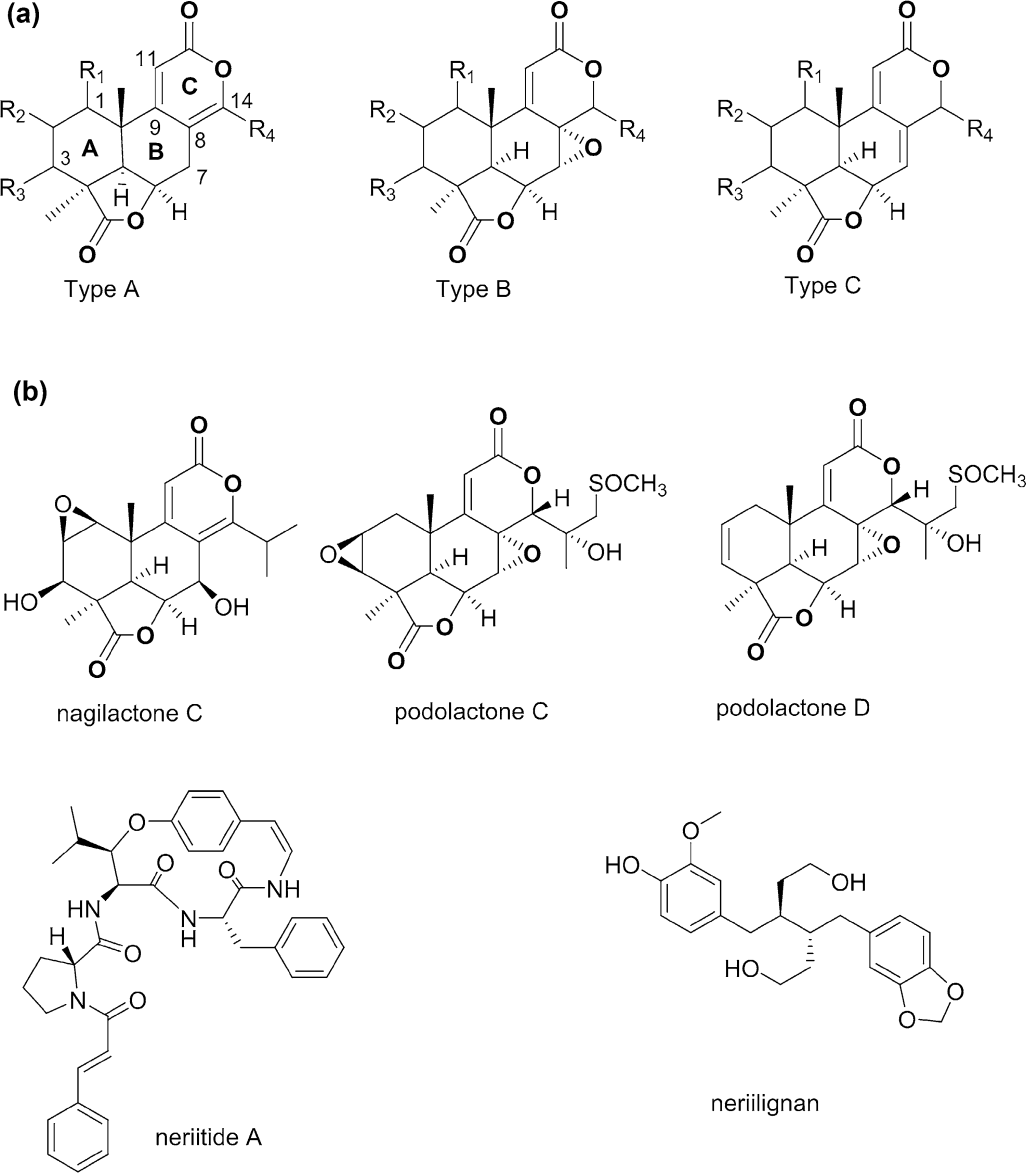
**a** Podolactone classification and **b** examples of previously isolated compounds from *P. neriifolius*

*Podocarpus neriifolius* D. Don (Podocarpaceae) is a tree growing in south Asian countries, such as Nepal and Vietnam, in Eastern China, and in the Pacific Islands [1]. While the wood of this plant is used as timber for furniture and in paper-making, its edible fruits are consumed raw or cooked, and decoctions from its leaves are used in folk medicine to relieve rheumatism and painful joints [2]. Plants of the genus *Podocarpus* have been reported to exhibit a variety of biological activities ranging from plant-growth regulation [3, 4] to antibacterial [5] and antiproliferative [6–9] effects. These activities have been attributed mainly to their chemotaxonomic markers, the nor- and bisnorditerpene dilactones, referred to as the podolactones or nagilactones [10, 11]. The podolactones exist in three main classes (types A-C) depending on the conjugated system between the B and C rings. As such, type A possesses a [8(14), 9(11)-dienolide] moiety, while types B and C are characterized by the presence of [7*α*,8*α*-epoxy-9(11)-enolide] and [7(8), 9(11)-dienolide] groups, respectively (Fig. 1) [7, 10, 12]. *P. neriifolius* produces several podolactones including the cytotoxic nagilactone C [13], the sulfur containing derivatives, podolactones C and D [14–16], and more recently a new cyclopeptide, neriitide A and the lignan, neriilignan, were reported from the leaves of this plant [17] (Fig. 1). In a continuing effort to discover potential lead anticancer agents from natural sources as part of a multidisciplinary program project grant [18], a root sample of *P. neriifolius* was investigated, resulting in the purification of one new (**2**) and three known (**1**, **3** and **4**) B-type podolactones, as well as three known totarane-type diterpenes (**5–7**). The isolation and structure determination of the obtained isolates, along with their antiproliferative properties against a panel of four human cancer cell lines (ovarian, breast, colon, and melanoma) are reported herein. Moreover, for the first time, in vivo evaluation of the major isolate, inumakilactone A (**3**), in a murine hollow fiber assay was conducted and described in the present study.

## Results and Discussion

**Fig. 2:**
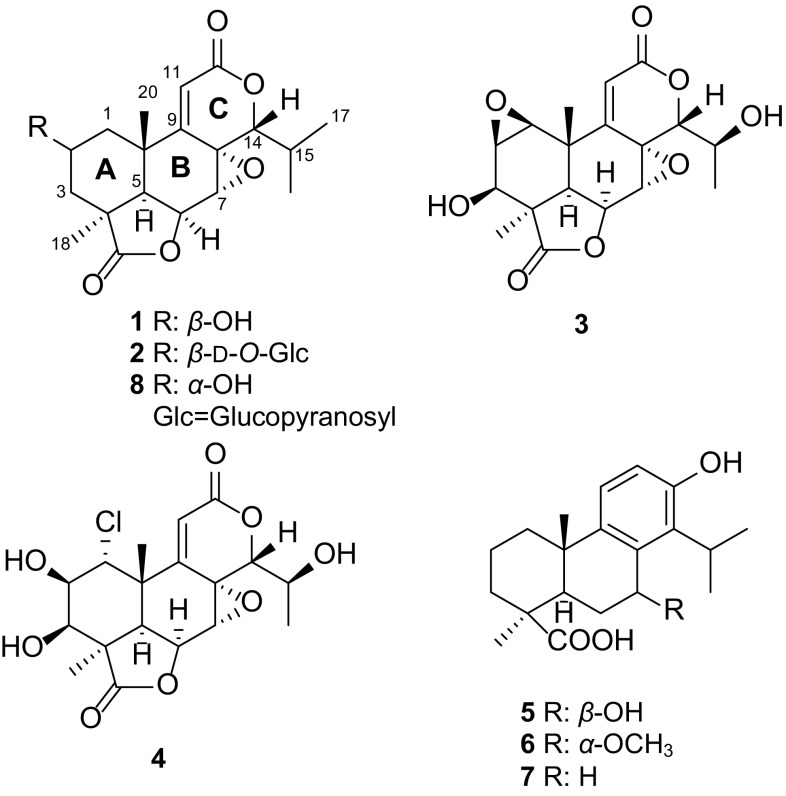
Structures of isolated compounds (**1**–**8**)

The bioactivity-guided fractionation of the cytotoxic ethyl acetate-soluble extract (IC_50_ = 4.3 µg/mL against the HT-29 human colon cancer cells) from the root sample of *Podocarpus neriifolius* led to the isolation of seven compounds including a new B-type podolactone glucoside (**2**) and six known diterpenoids (**1**, **3–7**), which were identified by spectroscopic data interpretation and comparison with published values (Fig. 2).

Compound **1** was obtained as a white amorphous powder, and its positive HRESIMS displayed a sodiated molecular ion peak at *m/z* 371.1469, equivalent to C_19_H_24_NaO_6_^+^ (calcd. for C_19_H_24_NaO_6_^+^, 371.1465). Preliminary interpretation of its 1D and 2D NMR spectroscopic data led to the assignment of its corresponding planar structure as a B-type podolactone [10, 19]. A subsequent literature search for this chemical structure revealed that the collected ^1^H and ^13^C NMR data for **1** matched with those of 3-deoxy-2*α*-hydroxynagilactone E (**8**), previously isolated from *Podocarpus nagi* [20]. However, careful inspection of the ^1^H NMR data measured in methanol-*d*_4_ revealed that the splitting pattern and coupling constants (*J)* values of H-2 (dddd, *J* = 13.5, 8.5, 7.4, 4.9 Hz) shared a close similarity with those of the revised C-type podolactone 2*β*-hydroxynagilactone F (H-2, dddd, *J* = 12.9, 9.1, 7.2, 5.1 Hz) [21], and suggested a comparable 2*β* configuration of the hydroxy group substitution at C-2. A very recent study on *P. nagi* reporting the X-ray structure of 3-deoxy-2*β*-hydroxynagilactone E confirmed this assertion [11]. While this compound was reported as a new molecule in this latter publication, its NMR data matched both the initial report [20], compound **8**, and the present data (Tables 1 and 2). Thus the structure of compound **8** as reported therein [20] should be revised as 3-deoxy-2*β*-hydroxynagilactone E (**1**).

**Table 1:** ^1^H NMR data for **1**, **2**, and **8** (in pyridine-*d*_*5*_, 400 MHz, *δ* in ppm, *J* in Hz)

Position	**1** (present study)	**1** [11]	**8** [20]	**2**
**1a**	2.42 (overlap, dd, 13.5, 8.5)	2.47 (dd, 13.5, 8.7)	2.42 (dd, 13.2, 9.1)	2.36 (dd, 13.8, 8.7)
**1b**	1.80 (dd, 13.5, 7.4)	1.84 (dd, 13.5, 7.5)	1.81 (dd, 13.2, 7.3)	1.81 (dd, 13.8, 7.6)
**2**	4.20 (m)^a^	4.24 (m)	4.22 (m)	4.30 (overlap, m)
**3a**	2.14 (dd, 13.5, 4.9)	2.19 (dd, 13.7, 4.8)	2.15 (dd, 13.6, 4.4)	2.29 (overlap)
**3b**	2.48 (overlap, t, 13.5)	2.52 (t, 13.7)	2.48 (dd, 13.6, 13.2)	2.31 (overlap)
**4**				
**5**	1.95 (d, 5.0)	1.99 (d, 5.0)	1.96 (d, 4.0)	1.77 (d, 4.9)
**6**	5.14 (dd, 5.0, 1.4)	5.18 (dd, 5.0, 1.5)	5.13 (d, 4.0)	5.07 (dd 4.9, 1.3)
**7**	4.24 (d, 1.4)	4.28 (d, 1.5)	4.23 (s)	4.2 (d, 1.3)
**8**				
**9**				
**10**				
**11**	6.30 (s)	6.35 (s)	6.30 (s)	6.17 (s)
**12**				
**14**	4.58 (d, 3.3)	4.62 (d, 3.3)	4.58 (d, 2.0)	4.51 (d, 3.3)
**15**	1.96 (overlap, m)	1.98 (m)	1.97 (m)	1.93 (m)
**16**	1.16 (d, 6.7)	1.20 (d, 6.7)	1.18 (d, 6.6)	1.14 (d, 6.7)
**17**	1.02 (d, 6.7)	1.05 (d, 6.7)	1.03 (d, 6.6)	1.00 (d, 6.7)
**18**	1.31 (s)	1.34 (s)	1.32 (s)	1.25 (s)
**19**				
**20**	1.28 (s)	1.31 (s)	1.30 (s)	1.13 (s)
**1′ (Glc-1)**				4.99 (d, 7.7)
**2′ (Glc-2)**				4.07 (t, 8.2)
**3′ (Glc-3)**				4.27 (overlap)
**4′ (Glc-4)**				4.24 (overlap)
**5′ (Glc-5)**				3.99 (ddd, 8.7, 5.8, 2.2)
**6′ (Glc-6)**				4.36 (dd, 11.6, 5.8)
				4.60 (br d, 11.6)

^a^Appeared as “dddd” (13.5, 8.5, 7.4, 4.9) in CD_3_OD

**Table 2:** ^13^C NMR data for **1**, **2**, and **8** (in pyridine-*d*_*5*_, 100 MHz, *δ* in ppm)

Position	**1** (present study)	**1** [11]	**8** [20]	**2**
**1**	40.4	41.1	40.65	38.4
**2**	63.5	64.2	63.70	71.4
**3**	38.3	39.0	38.52	34.3
**4**	42.0	42.8	42.32	41.7
**5**	42.1	42.7	42.32	41.9
**6**	72.8	73.5	73.03	72.7
**7**	54.1	54.8	54.33	54.0
**8**	57.8	58.5	58.00	58.0
**9**	159.1	159.8	159.34	158.6
**10**	36.7	37.5	37.01	36.5
**11**	118.2	118.9	118.44	118.3
**12**	163.6^a^	164.4	163.80	163.5
**14**	82.6^a^	83.2	82.83	82.5
**15**	26.7	27.4	26.98	26.7
**16**	21.1	21.8	21.32	21.1
**17**	16.3	17.0	16.51	16.2
**18**	22.7	23.4	28.80	22.4
**19**	181.1^a^	181.8	181.30	180.9
**20**	28.6	29.3	22.95	28.3
**1′**				103.1
**2′**				75.1
**3′**				78.4
**4′**				71.6
**5′**				78.5
**6′**				62.7

^a^Deduced from HMBC correlation

Compound **2** was isolated as a white amorphous solid. Its HRESIMS gave a protonated molecular ion peak at *m/z* 511.2172 [M + H]^+^ indicating a molecular formula of C_25_H_34_O_11_ (calcd. for C_25_H_35_O_11_^+^, 511.2174). Inspection of the ^1^H and ^13^C NMR data revealed typical podolactone signals similar to those of **1** (Tables 1 and 2). For instance, in the ^1^H NMR spectrum, signals resonating at *δ*_H_ 6.17 (s), 5.07 (dd, *J* = 1.2, 5.0 Hz), 4.20 (d, *J* = 1.2 Hz), and 1.77 (d, *J* = 5.0 Hz) could be attributed to the protons H-11, H-6, H-7, and H-5, respectively. Furthermore, ^13^C NMR resonances at *δ*_C_ 180.9 (C-19) and 163.5 (C-12) corresponding to *γ* and *δ*-lactone carbonyls, respectively, along with the signals at *δ*_C_ 158.6 (C-9), 118.3 (C-11), 57.7 (C-8), and 54.0 (C-7) distinctive of a conjugated 7*α*,8*α*-epoxy-9(11)-enolide structural unit, suggested that this compound has a B-type podolactone core [12, 19]. In addition, a beta-anomeric proton at H-1′, *δ*_H_ 4.99 (*J* = 7.8 Hz) and six ^13^C NMR signals resonating at *δ*_C_ 103.1, 75.1, 78.4, 71.6, 78.5, and 62.7 indicated a *β*-glucopyranosyl moiety [11]. These observations were corroborated by the presence of a fragment peak [M−162]^+^ at 349 in the MS/MS spectrum, suggesting the loss of a glucopyranosyl unit (Electronic supplementary material). Comparison of the ^1^H and ^13^C NMR data of the glucopyranosyl unit with those of previously isolated podolactone glucosides [11, 22] further confirmed the nature of the saccharide.

**Fig. 3:**
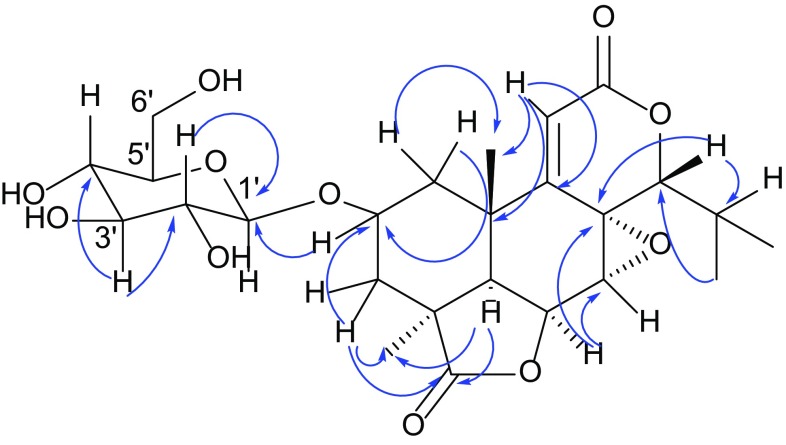
Key HMBC correlations for **2**

Moreover, 20 carbon signals corresponding to the 2-*O*-substituted type-B podolactone were observed in the ^13^C NMR spectrum (Table 2). Accordingly, compound **2** could be assigned as a glucosylated B-type podolactone with a glucopyranosyl moiety attached either to the A ring or at C-14, since C-6, C-7, and C-11 were protonated. However, the presence of signals at *δ*_H_ 1.14 (d, *J* = 6.7 Hz), 1.00 (d, *J* = 6.7 Hz), and 1.93 (m) corresponding to an isopropyl side chain at C-14, indicated that the glucose unit must be linked to the A ring of the podolactone core (Fig. 1, Tables 1 and 2). Comparison of the ^13^C NMR spectroscopic data of **1** and **2** showed a *β*-d-glycosylation shift of +8 ppm at C-2 (63.5 ppm in **1** *vs.* 71.4 ppm in **2**), and further shift values of − 2.0 and − 3.9 ppm for C-1 (40.4 ppm in **1** to 38.4 ppm in **2**) and C-3 (38.3 ppm in **1**  to 34. 3 ppm in **2**), respectively [23]. Key HMBC correlations, including a cross-peak between H-2 and C-1′ confirmed the above conclusions on the attachment of the *β*-d-glucopyranosyl unit at C-2 (Fig. 3). Thus, the structure of compound **2** was assigned as a C-2 glycosylated derivative of **1**, named nagilactone G-2*β*-*O*-*β*-d-glucoside.

In addition to compounds **1** and **2**, two known B-type podolactones, inumakilactone A (**3**) [8] and makilactone E (**4**) [19], along with three known totarane-type diterpenes, inumakiols D (**5**) and E (**6**), and 4*β*-carboxy-19-nor-totarol (**7**) [8] were isolated and identified during the present study.

All these isolates were evaluated for their antiproliferative activity against four human cancer cell lines, namely, HT-29 (colon), MDA-MB-231 (breast), OVCAR3 (ovarian), and MDA-MB-435 (melanoma). Compounds **1** and **3** exhibited moderate potency across all four cell lines (Table 3), whereas the remaining compounds were inactive (IC_50_ > 10 μM). The antiproliferative activity of the podolactones isolated was consistent with the reported SAR studies where the aglycones but not their glucoside derivatives proved to be active [7, 11]. Inumakilactone A (**3**) which was isolated in a larger quantity than **1**, was further assessed for its in vivo antitumor efficacy in a hollow fiber assay using the three human cancer cell lines, HT-29, MDA-MB-435, and OVCAR3 [24, 25]. Test mice within the treatment groups were initially administered 1 and 2 mg/kg doses of **3**, but following signs of toxicity, the dosages were reduced to half in each group. Nevertheless, compound **3** did not display any significant effect on cell survival for all three cell lines tested when compared to the vehicle control. While inumakilactone A (**3**) has been previously reported as an antiproliferative compound in vitro [7], this is the first report of its in vivo evaluation in the murine hollow fiber model using the above-mentioned human cancer cell lines.

**Table 3:** Cytotoxicity data (IC_50_, μM) for **1** and **3** against a panel of four human cancer cell lines

Compound	**HT-29** (colon)	**MDA-MB-435** (melanoma)	**MDA-MB-231** (breast)	**OVCAR3** (ovarian)
**1**	6.3	2.4	4.2	2.9
**3**	> 10	3.7	6.6	5.2
Vinblastine (nM) (positive control)	4.52	0.49	8.78	1.82

## Experimental

### General Experimental Procedures

The optical rotation was measured on a modular circular polarimeter (MCP) 150 (software version 1.50; Anton Paar OptoTec GmbH, Seelze-Letter, Germany). A Hitachi U-2910 spectrophotometer (Hitachi High-Technologies Corporation, Tokyo, Japan) was utilized to obtain UV/vis data. High-resolution mass spectra were collected with a LTQ Orbitrap™ (Thermo Fisher Scientific Inc., Bremen, Germany) equipped with ITMS and FTMS analyzers, covering a mass range of *m/z* 50-4000, and with resolution ranging from 7500-100,000, operated in the positive-ion mode using sodium iodide for calibration, as well as with a 15T Bruker FT-ICR mass spectrometer. NMR spectroscopic data were recorded with a Bruker AVIII 400 HD NMR spectrometer (Bruker Biospin Billerica, MA, USA) at room temperature (298–300°K). High-performance liquid chromatography was performed with a Hitachi Primaide HPLC apparatus (Hitachi High-Technologies Corporation, Tokyo, Japan), equipped with a Primaide 1110 pump with a degasser, a Primaide 1210 autosampler, and Primaide 1430 diode array detector, and a semi-preparative C_18_ HPLC column (Dynamax and Cogent 250 mm × 10 mm i.d.). Column chromatography was conducted using Sephadex^®^ LH-20 resin (Supelco, Bellefonte, PA, USA), normal-phase silica gel [(40–63 µm particle; 230 × 400 mesh) (Sorbent Technologies, Atlanta, GA, USA)] and reversed-phase C_18_ silica gel (Sorbent Technologies, Atlanta, GA, USA). Analytical thin-layer chromatography (TLC) was performed on precoated (200 µm normal phase, and 150 µm, reversed-phase C_18_) aluminum-backed silica gel plates supplemented with fluorescence indicator (UV light at 254 nm) (Sorbent Technologies, Atlanta, GA, USA). Solvents used for chromatographic separations and spectrometric analysis (ACS, HPLC and MS-grade) were purchased from Fisher Scientific (Fair Lawn, NJ, USA). Deuterated solvents for NMR were purchased from Cambridge Isotope Laboratories, Inc. (Andover, MA, USA) and Sigma Aldrich (St Louis, MO, USA).

### Plant Material

The roots of *P. neriifolius* D. Don (Podocarpaceae) were collected during the period July–August 2011 in Cotuy forest at the Nui Chua National Park, Ninh Thuan Province, Vietnam (11^o^ 43.159′ N; 109^o^ 08.208′ E.) and identified by Dr. D. Doel Soejarto (College of Pharmacy, University of Illinois at Chicago; Field Museum of Natural History, Chicago, IL). A voucher specimen (collection number: DDS 14601) was deposited at the John G. Searle Herbarium of the Field Museum of Natural History, Chicago, IL, USA, under accession number F-2294563.

### Extraction, Isolation, and Structure Determination

The air-dried powdered root sample (100 g) of *P. neriifolius* was extracted by exhaustive percolation in methanol. Evaporation of this percolate *in vacuo* resulted in a crude MeOH extract (6.1 g), which was re-suspended in a hydromethanolic solution and further partitioned with hexanes and subsequently with EtOAc. The three obtained extracts, namely, hexanes (D1, 296 mg), aqueous (D2, 3.0 g), and EtOAc (D3, 2.6 g) partitions were evaluated for their cytotoxicity in vitro, and the active (IC_50_ < 20 µg/mL) EtOAc partition in having exhibited an IC_50_ value of 4.3 µg/mL was further purified. An initial aliquot of the ethyl acetate-soluble fraction (608 mg) was applied to a Sephadex LH-20 gel column (41 cm × 1.3 cm) and eluted with CH_2_Cl_2_-MeOH (1:1 v/v), affording six fractions F1-F6. Fraction F3 (ca. 192 mg) was further chromatographed on a normal-phase silica gel column (30 cm × 1.2 cm) with CHCl_3_-EtOAc–MeOH (7:2:1 v/v) into five sub-fractions, F3.1-F3.5. Fractions F3.2 and F3.3 afforded compounds **4** (2.4 mg) and **3** (1.6 mg) as precipitated solids, respectively. Another batch of the EtOAc extract (1.0 g) was also subjected to separation on a Sephadex LH-20 column (41 cm × 1.3 cm) using CH_2_Cl_2_-MeOH (1:1 v/v) for elution, affording six fractions, F1′–F6′. Fraction F3′ (327.1 mg, IC_50_ = 3.8 µg/mL) was then purified on normal-phase silica gel with CHCl_3_-EtOAc-MeOH (7:2:1 v/v) into eight sub-fractions. Fractions F3′.3 (46 mg) and F3′.4 (20.7 mg) with IC_50_ values of 0.4 and 0.5 µg/mL, respectively, both afforded compound **3** (inumakilactone A, 12.3 mg total) as a precipitated product. Fraction F3′.2 (131.5 mg, IC_50_ = 15.1 µg/mL) formed a white crystalline precipitate (**4**, 9.7 mg), and its supernatant was further chromatographed on a reversed-phase C_18_ column into three sub-fractions (F3′.2.1–F3′.2.3), eluted with 40, 70, and 100% MeOH/H_2_O, respectively. Semi-preparative HPLC purification of F3′.2.1 (IC_50_ = 2.1 µg/mL) on C_18_ in CH_3_CN/H_2_O with an increasing gradient (30:70 to 40:60 in 10 min, 40:60 to 100:0 in 12 min, 100:0 for 5 min) and at a detection wavelength of 234 nm, afforded compounds **1** (3-deoxy-2*β*-hydroxynagilactone E, 2.1 mg) and **5** (inumakiol D, 1.4 mg), eluted at 19.1 and 20.1 min, respectively. Similarly, fraction F3′.2.2 (IC_50_ = 7.8 µg/mL) was injected onto a C_18_ semi-preparative HPLC column and eluted with CH_3_CN/H_2_O (50:50 to 70:30 in 10 min, 70:30 to 85:15 in 6 min, 85:15 to 100:0 in 1 min, 100:0 for 3 min, 100:0 to 50:50 in 2 min, 50:50 for 5 min, at 210 nm), and also afforded compound **1** (t_R_ = 8.7 min, 1.2 mg) along with inumakiol E (**6**, t_R_ = 15.4 min, 5.0 mg) and 4*β*-carboxy-19-nor-totarol (**7**, t_R_ = 19 min, 0.6 mg). Fraction F2′ (329 mg, IC_50_ = 7.0 µg/mL) was pre-fractionated on C_18_ column using MeOH/H_2_O (70:30 and 100:0 v/v) into two sub-fractions. Of these, fraction F2′.1 was further separated on a C_18_ semi-preparative HPLC column in CH_3_CN/H_2_O (20:80 to 50:50 in 20 min, 50:50 to 100:0 in 1 min, 100:0 for 5 min, at 234 nm) affording compounds **2** (t_R_ = 16.2 min, 2.5 mg) and **1** (t_R_ = 22.2 min, 1.5 mg). The structure of each isolate was determined by comprehensive spectroscopic (1D and 2D NMR, HRESIMS) data analysis coupled with comparison with the published phytochemical literature.

**Nagilactone G-2*β*-*O*-*β*-d-glucoside (2):** [*α*]_*D*_^20^ +31 (*c* .10, MeOH); UV (H_2_O) λ_max_ (logε) 201 (3.83), 279.5 (2.87) nm; ^1^H and ^13^C NMR data shown in Tables 1 and 2; HRESIMS *m/z* 511.2172 [M + H]^+^ (calcd for C_25_H_35_O_11_^+^, 511.2174).

### Antiproliferative Evaluation Using Cancer Cell Lines

Preliminary cytotoxicity screening of plant extracts against the human colon cancer cell line HT-29, and subsequent in vitro evaluation of the isolated compounds against four human cancer cell lines, including HT-29, MDA-MB-231 (breast), MDA-MB-435 (melanoma), and OVCAR3 (ovarian), were performed following previously reported protocols [25, 26].

### In Vivo Hollow Fiber Assay

Immunodeficient NCr *nu*/*nu* mice (7-weeks-old) were procured from Taconic Biosciences (Rensselaer, NY, USA) and housed in microisolation cages at room temperature and with a relative humidity of 50–60% under 12:12 h light–dark cycle. All animal procedures were performed following approval by the University of Illinois at Chicago (UIC) Animal Care and Use Committee (protocol number 16-035), and the mice were treated according to the institutional guidelines for animal care.

The antitumor activity of inumakilactone A (**3**) against OVCAR3, HT-29, and MDA-MB-435, was evaluated in vivo using an established hollow fiber assay procedure described previously [24, 25, 27]. Briefly, cells were first cultured in hollow fibers 2 days (OVCAR3 cells, 4 × 10^6^ cells/mL) and 1 day (HT-29, 1 × 10^6^ cells/mL and MDA-MB-435, 2.5 × 10^6^ cells/mL) prior to insertion. Inumakilactone A (**3**) was dissolved in DMSO and further diluted with 60% PEG 300 and 30% water. The immunodeficient NCr *nu*/*nu* mice were divided into four groups, including a paclitaxel positive control group (*n* = 2), a negative vehicle group (*n* = 6), and inumakilactone A (**3**) treatment groups receiving 1 mg/kg (*n* = 6) or 2 mg/kg (*n* = 3). On day 0, hollow fibers containing the human cancer cells were implanted in the abdominal cavity of the mice. The animals were then injected *i.p.* once daily for four days (day 3 through day 6) with vehicle, paclitaxel, or **3**. Each mouse was weighed daily during the study. Doses were reduced to half after one animal from each treatment group died and the rest exhibited signs of toxicity after the second injection. The remaining mice were sacrificed on day 7. The fibers were removed, and viable cell mass was measured by a modified MTT [3-(4,5-dimethylthiazol-2-yl)-2,5-diphenyltetrazolium bromide]. Statistical analysis was performed using ANOVA.

## Electronic supplementary material

Below is the link to the electronic supplementary material.

Supplementary material 1 (DOCX 1089 kb)
