# Supplementary material for: Bioactivity-Guided Isolation of Totarane-Derived Diterpenes from Podocarpus neriifolius and Structure Revision of 3-Deoxy-2α-hydroxynagilactone E
Source: Nat Prod Bioprospect. 2019 Feb 19;9(2):157–63. doi: 10.1007/s13659-019-0198-x (PMC6426912; doi:10.1007/s13659-019-0198-x)

**SUPPORTING INFORMATION**

**Bioactivity-guided isolation of totarane-derived diterpenes *Podocarpus neriifolius* and structure revision of 3-deoxy-2*α*-hydroxynagilactone E**

**P. Annécie Benatrehina^a^, Wei-Lun Chen^c^, Austin A. Czarnecki^c^, Hee-Byung Chai^a^, Daniel Levit^c^, Tran N. Ninh^e^, Xiaoli Zhang^b^, Djadja D. Soejarto^c,d^, Joanna E. Burdette^c^, A. Douglas Kinghorn^a^, and L. Harinantenaina Rakotondraibe^a,^***

**^a^**Division of Medicinal Chemistry and Pharmacognosy, College of Pharmacy, ^b^ Center for Biostatistics, The Ohio State University, Columbus, OH, USA

^c^College of Pharmacy, University of Illinois at Chicago, Chicago, IL, USA

^d^John G. Searle Herbarium of the Field Museum of Natural History, Chicago, IL, USA

^e^Institute of Ecology and Biological Resources, Vietnam Academy of Science and Technology Hanoi, Vietnam

*Corresponding author.

Tel: (614) 292-4733. Fax: (614) 292-2435. E-mail: rakotondraibe.1@osu.edu (L.H. Rakotondraibe)

**Table of content**

| **Figure** | **Content** | **Page** |
| --- | --- | --- |
| S1 | HRESIMS spectrum for **1** | 3 |
| S2 | ^1^H NMR spectrum of **1** measured in pyr-*d_5_* | 4 |
| S3 | ^13^C NMR spectrum of **1** measured in pyr-*d_5_* | 5 |
| S4 | HSQC of **1** measured in pyr-*d_5_* | 6 |
| S5 | HMBC of **1** measured in pyr-*d_5_* | 7 |
| S6 | ^1^H NMR spectrum of **1** measured in Met-*d_4_* | 8 |
| S7 | ^1^H NMR spectrum of **1** measured in Met-*d_4_* (cont’d) | 9 |
| S8 | HRESIMS spectrum for **2** | 10 |
| S9 | HRESIMS spectrum for **2** (MS/MS fragment) | 11 |
| S10 | ^1^H NMR of **2** measured in pyr-*d_5_* | 12 |
| S11 | Overlay ^1^H NMR spectra of **1** and **2** measured in pyr-*d_5_* | 13 |
| S12 | ^1^H NMR spectrum of **2** measured in Met-*d_4_* | 14 |
| S13 | Overlay ^1^H NMR spectra of **1** and **2** measured in Met-*d_4_* | 15 |
| S14 | ^13^C NMR of **2** measured in pyr-*d_5_* | 16 |
| S15 | HSQC spectrum of **2** measured in pyr-*d_5_* | 17 |
| S16 | HMBC spectrum of **2** measured in pyr-*d_5_* | 18 |

**Figure S1.** HRESIMS spectrum for **1**

*m/z* 371.1465 [M+Na]^+^, calcd C_19_H_24_NaO_6_^+^

**Figure S2.** ^1^H NMR spectrum of **1** measured in pyr-*d_5_*, 400 MHz


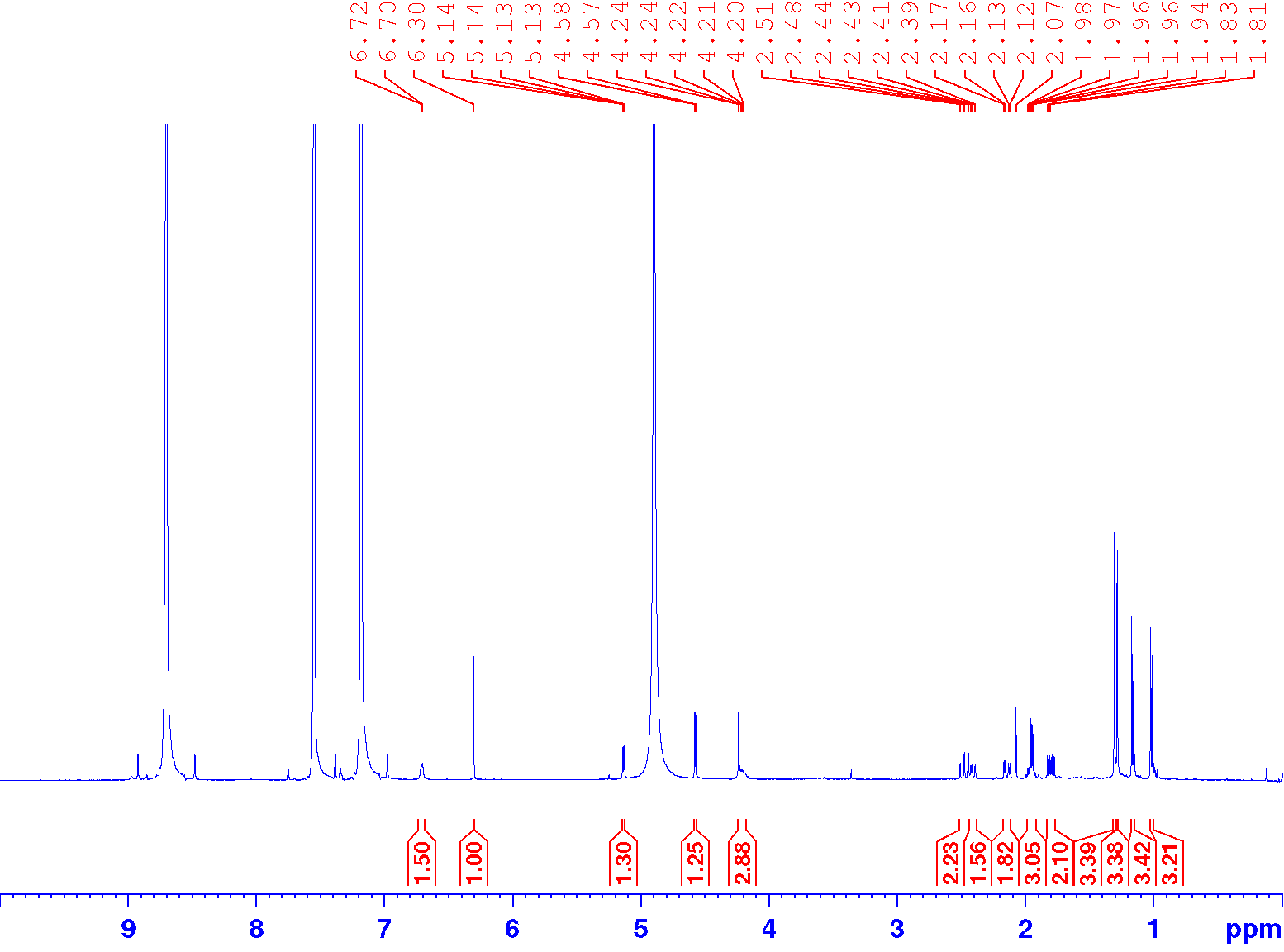


**Figure S3.** ^13^CNMR spectrum of **1** measured in pyr-*d_5_*, 100 MHz


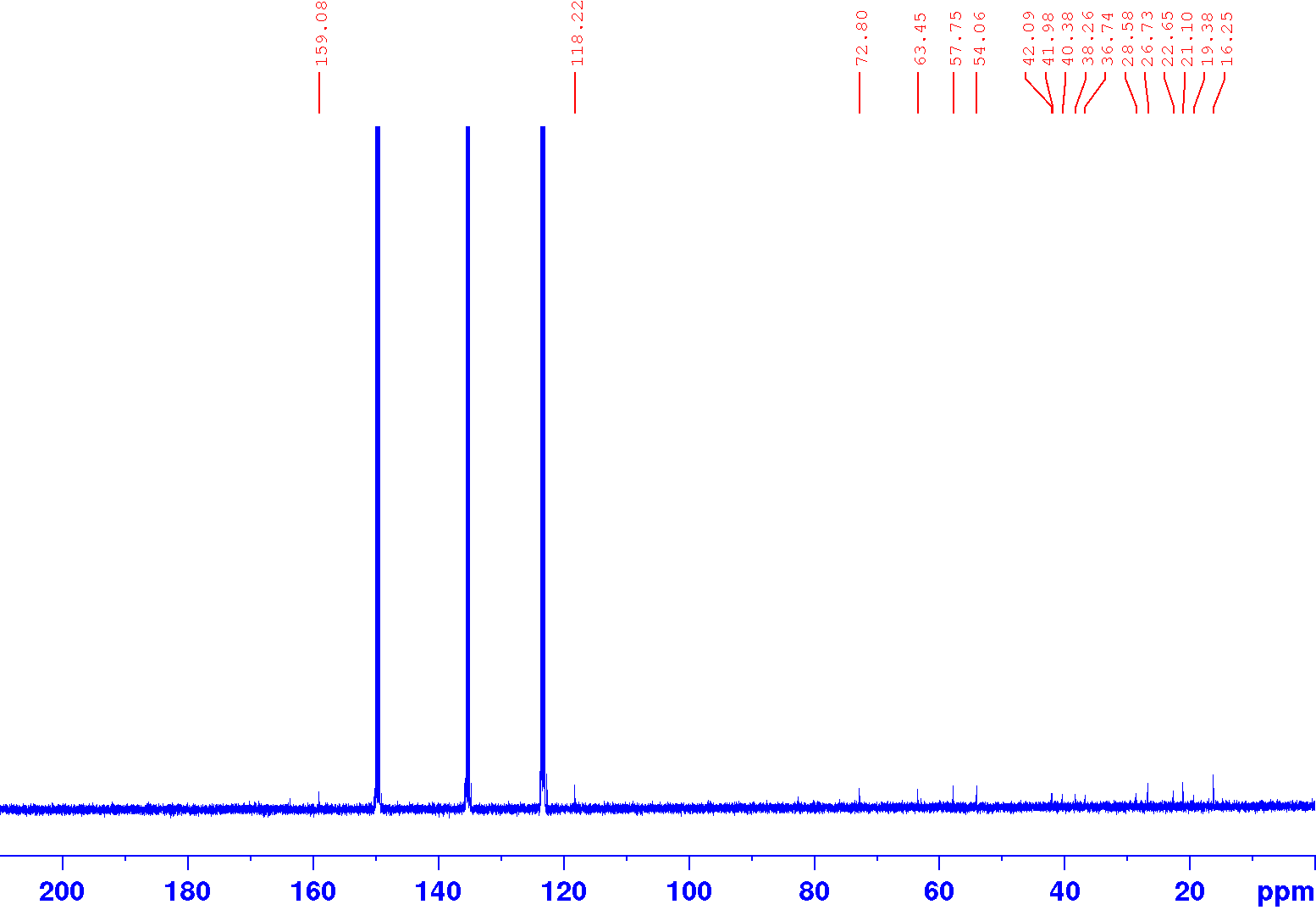


**Figure S4.** HSQC spectrum of **1** measured in pyr-*d_5_*


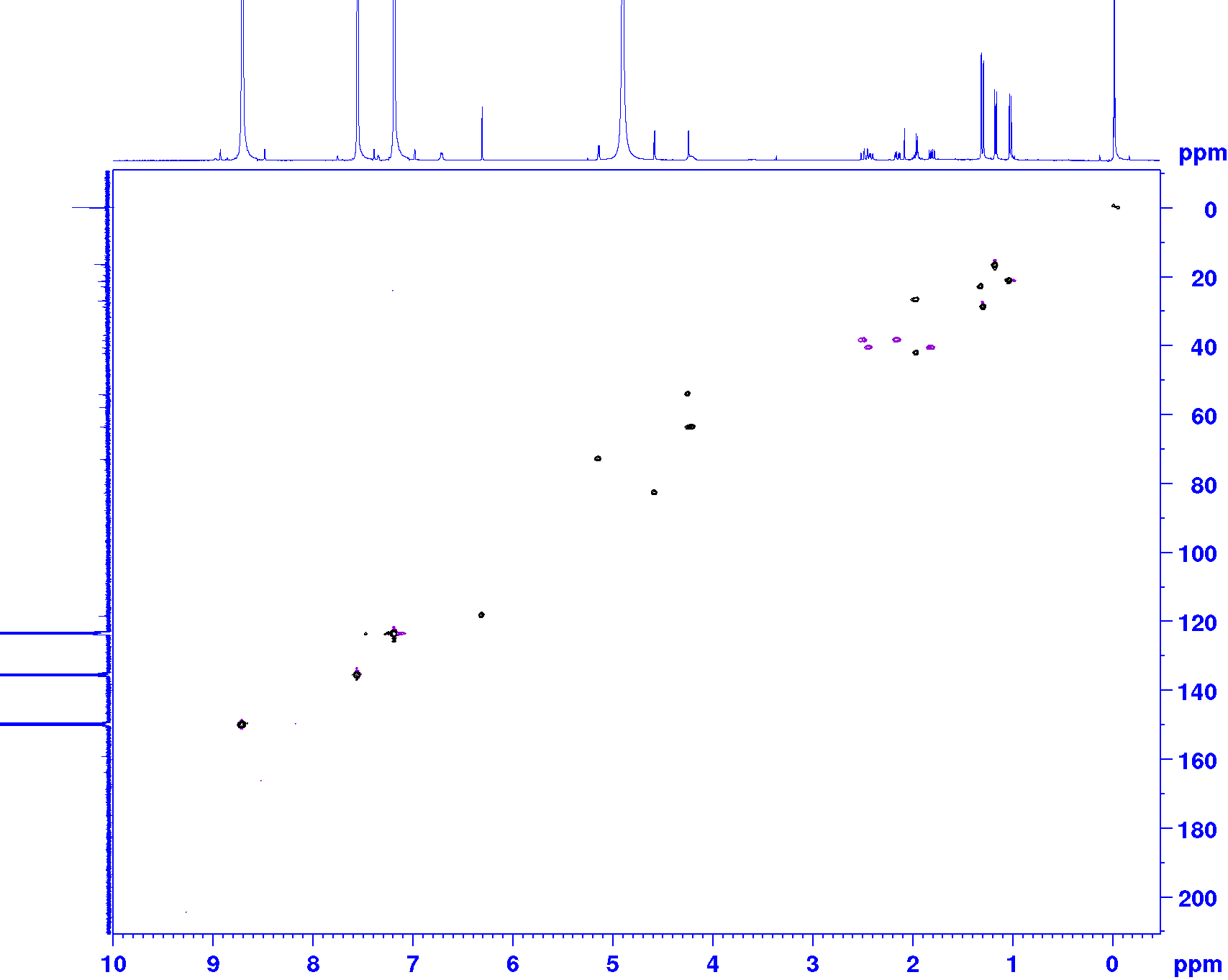


**Figure S5.** HMBC spectrum of **1** measured in pyr-*d_5_*


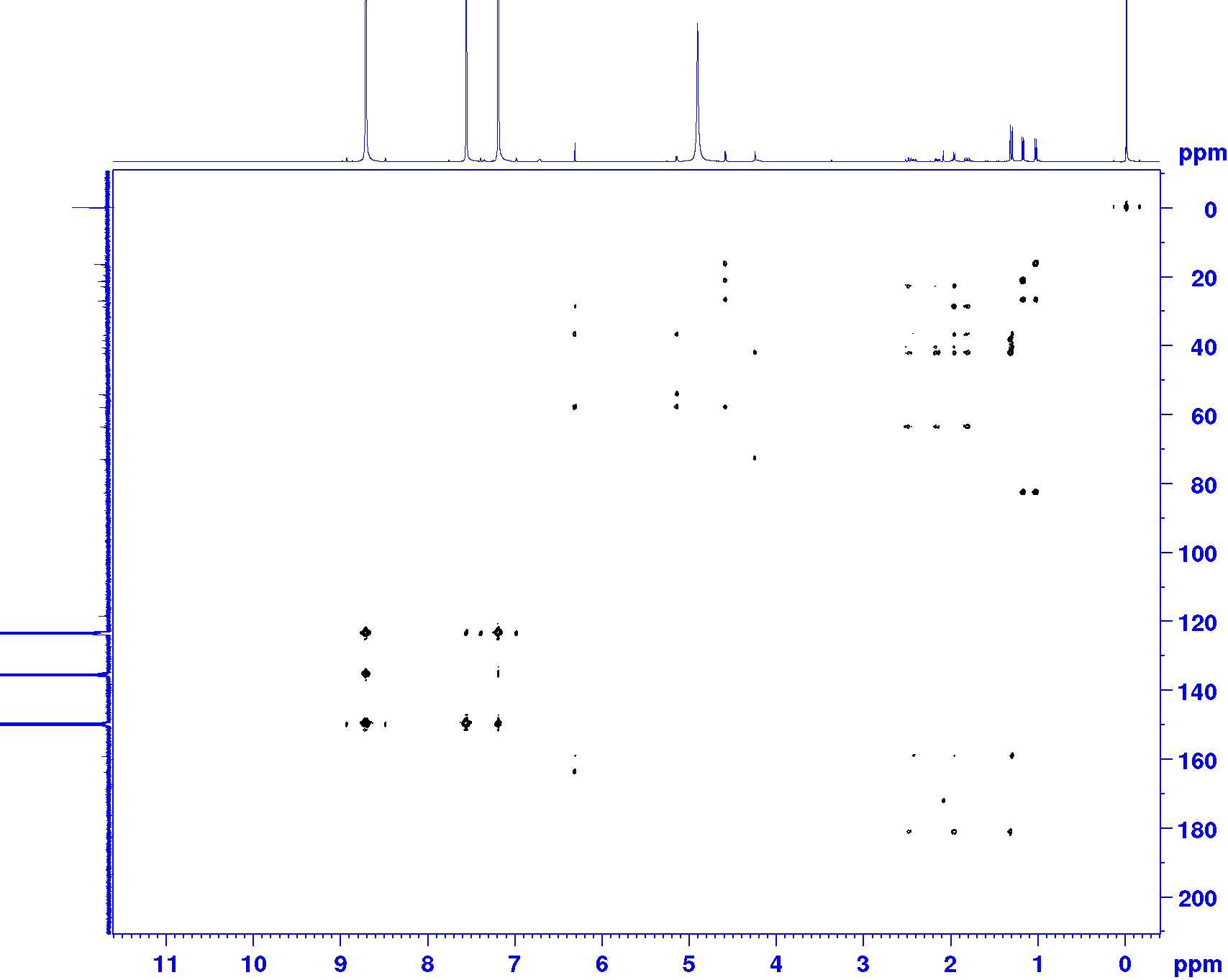


**
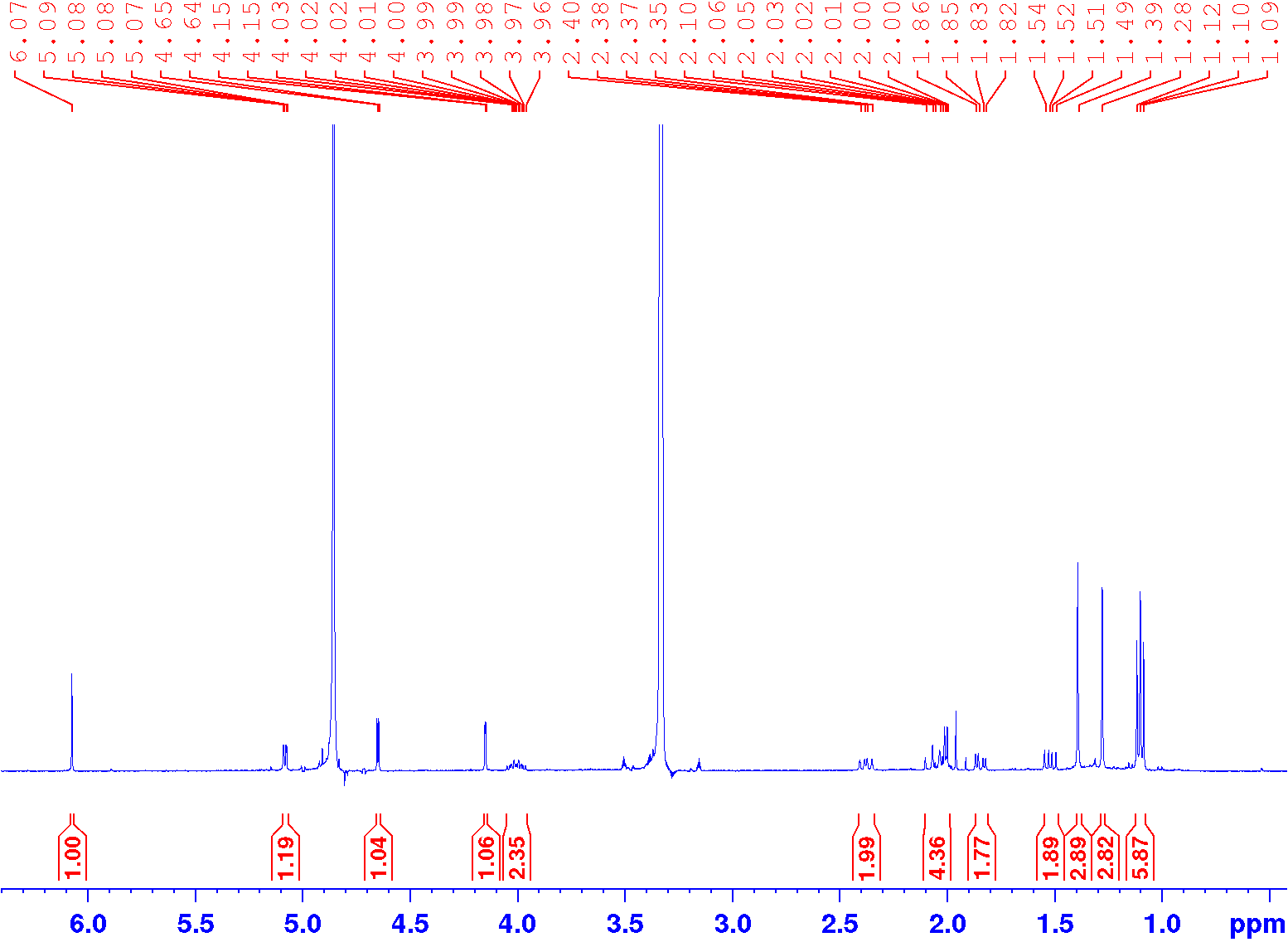
Figure S6.** ^1^H NMR spectrum of **1** measured in Met-*d_4_*, 400 MHz

**Figure S7.** ^1^H NMR spectrum of **1** measured in Met-*d_4_*, 400 MHz, cont’d


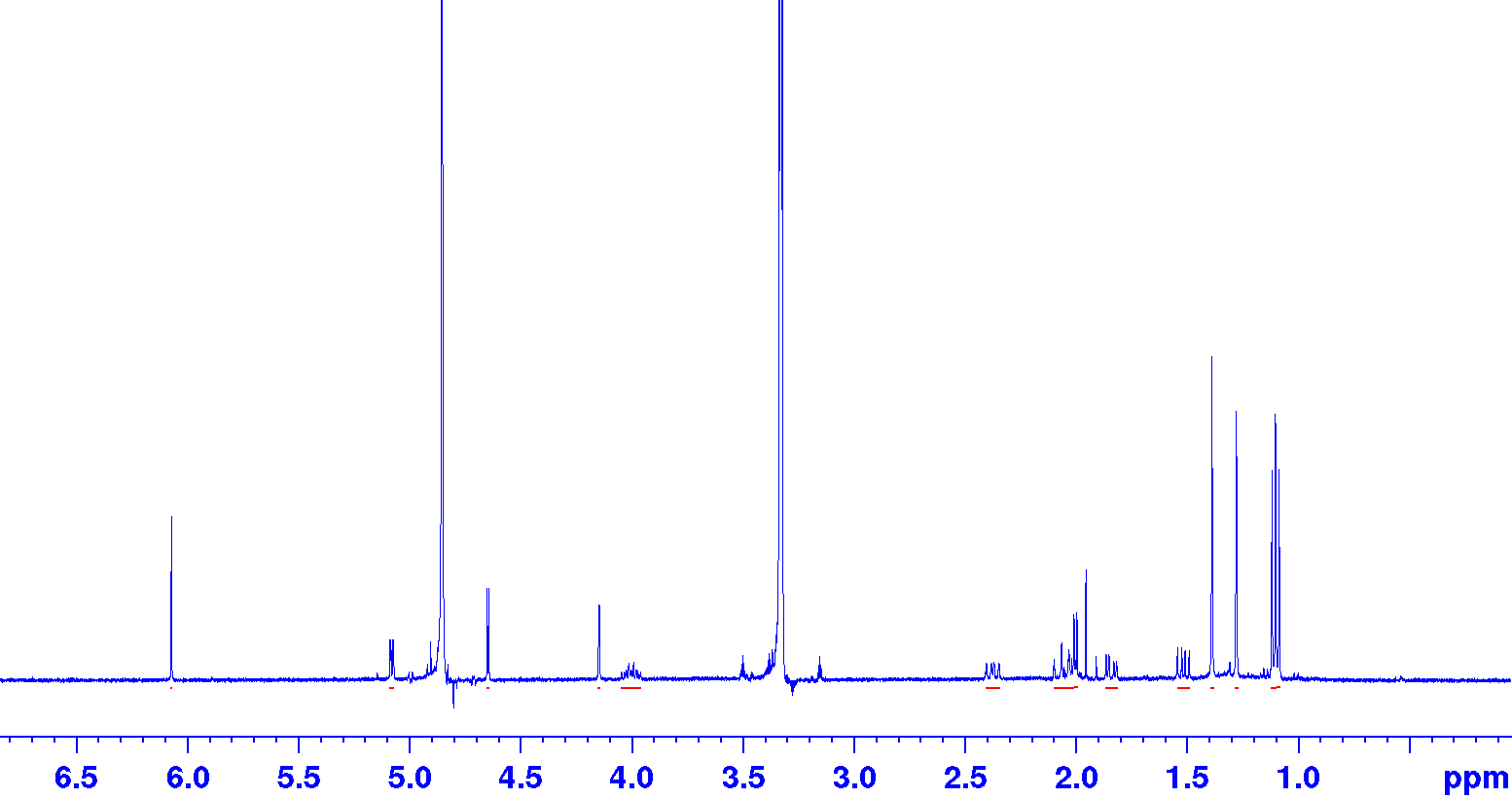

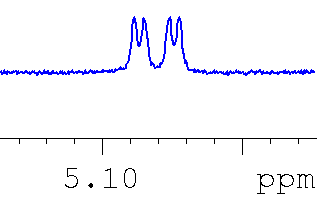

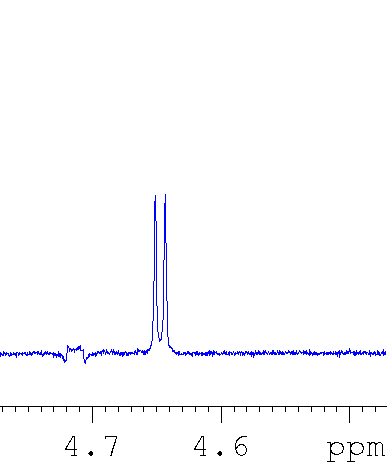


H-11

H-6

H-7


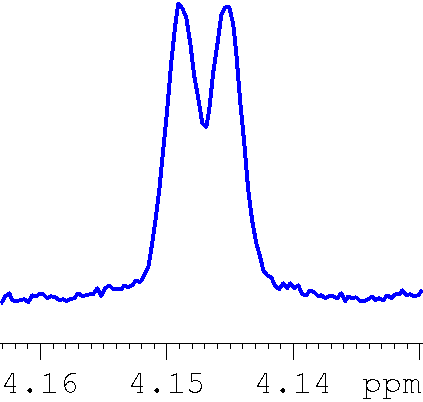


H-14

H-2


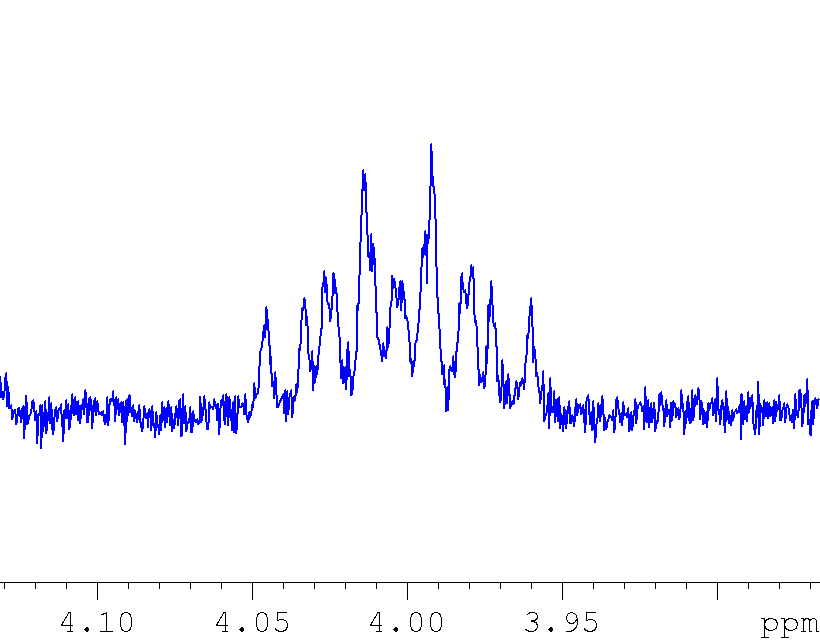

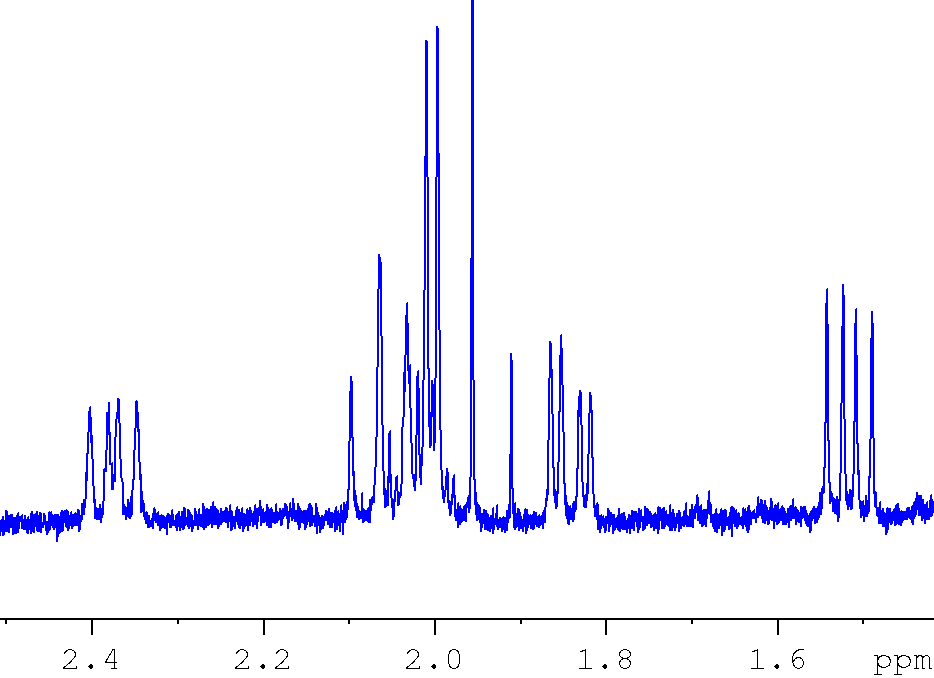

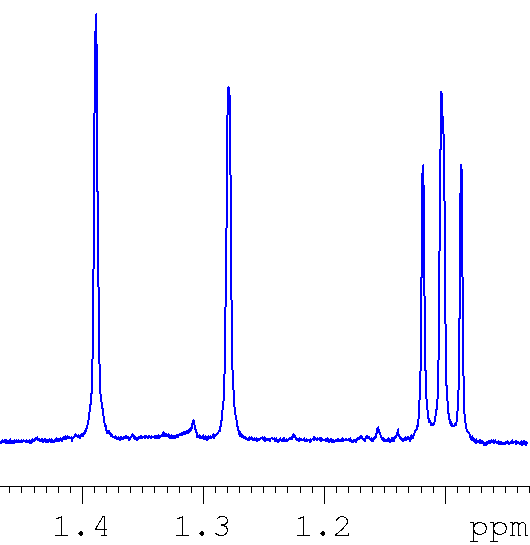


H-6

H-14

H-7

H-2

H-1a

H-1a

H-3a, H-15, H-5

H-1b

H-1b

H-3b

H-18

H-16, H-17

H-18

H-20

H-20

H-3a, H-15, H-5

H-3b

H-16, H-17

**Figure S8.** HRESIMS spectrum for **2**

**Figure S9.** HRESIMS spectrum for **2** (MS/MS fragment)

[M + H - 162.0527]

m/z 349.1645

[M+H]^+^

m/z 511.2172

**Figure S10.** ^1^H NMR spectra of **2** measured in pyr-*d_5_*, 400 MHz


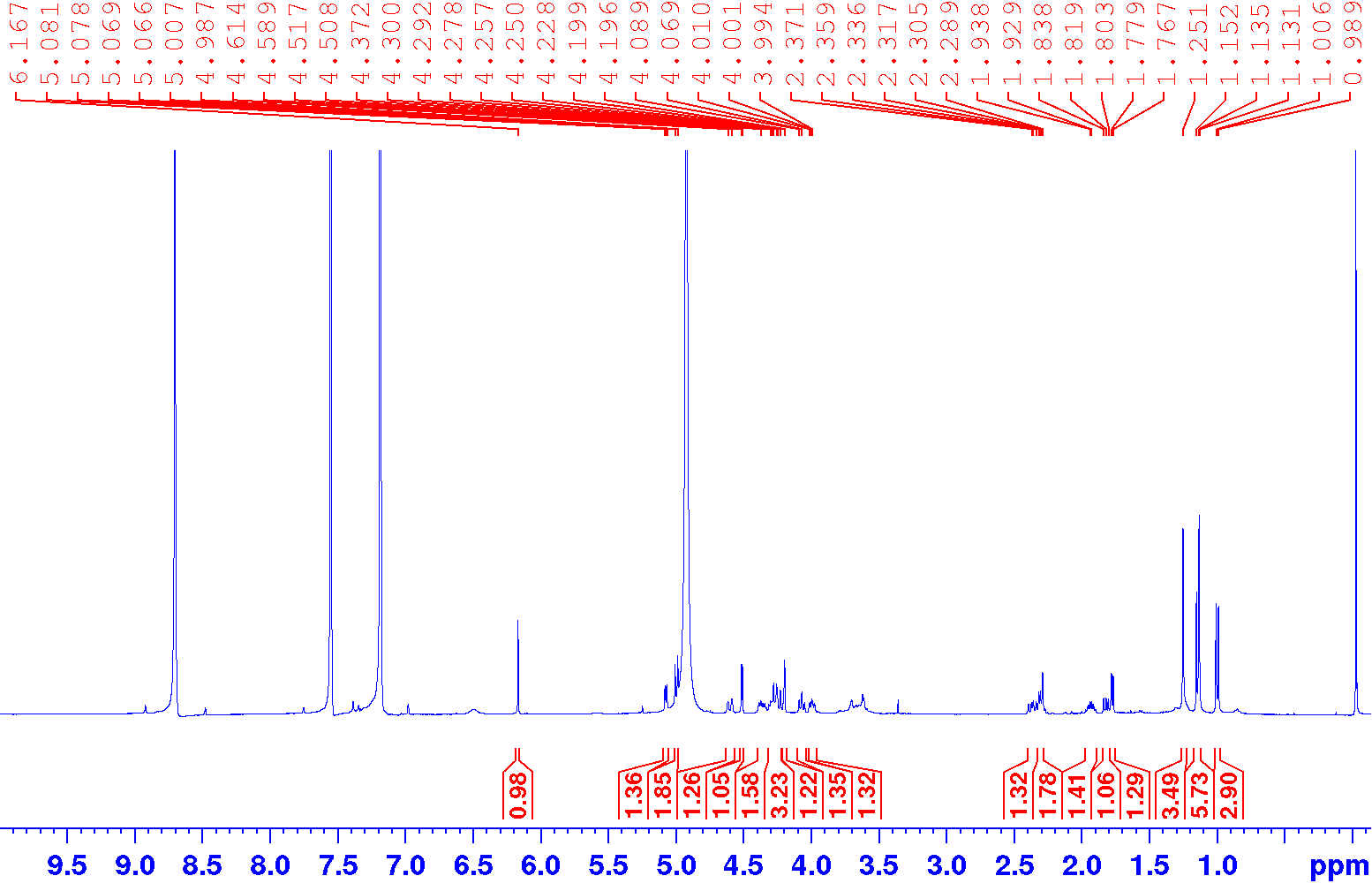

**Figure S11.** ^1^H NMR spectra of **1** (top) and **2** (bottom) measured in pyr-*d_5_*, 400 MHz


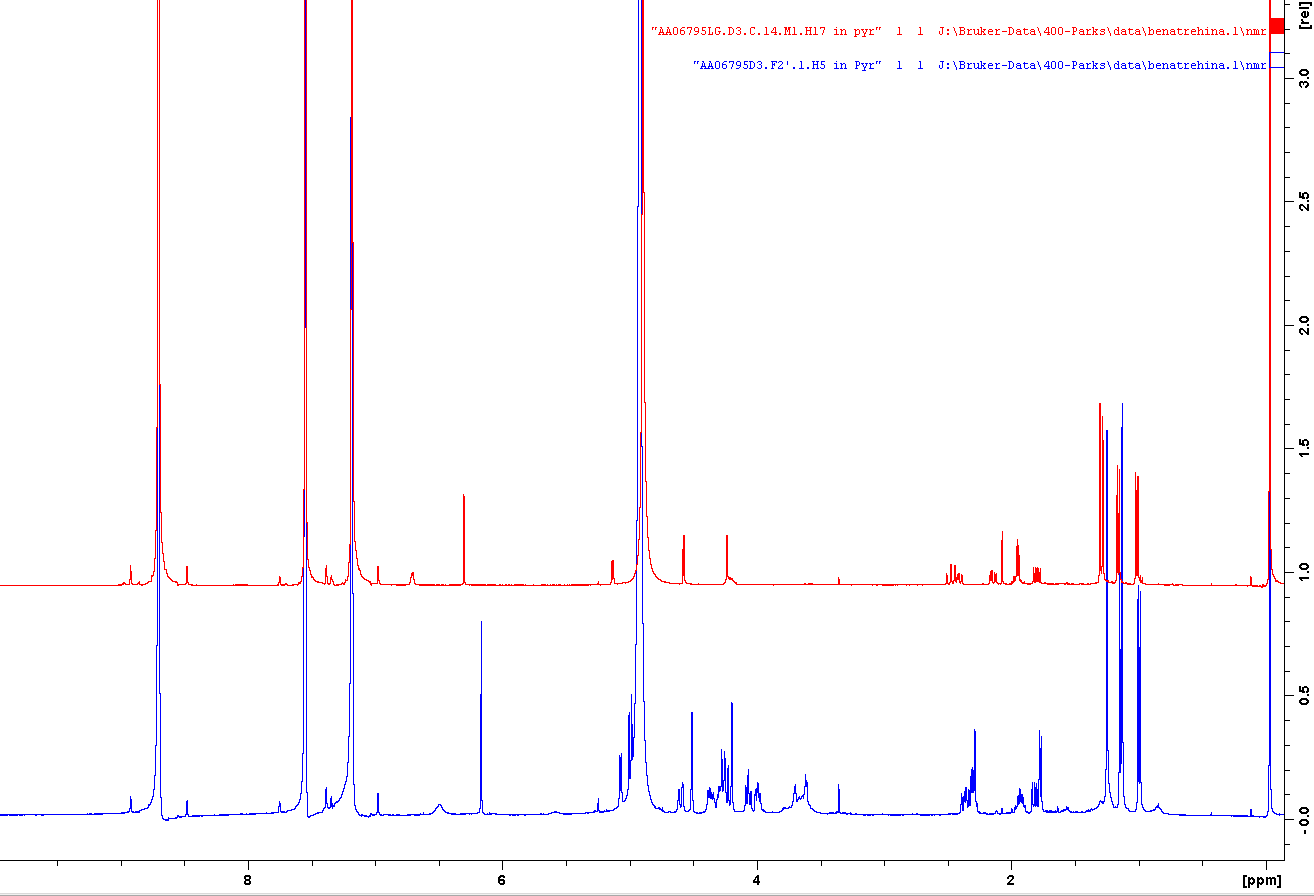

**1** R: *β*-OH

**2** R: *β*-*O*-glc

**Figure S12.** ^1^H NMR spectrum of **2** measured in Met-*d_4_*, 400 MHz


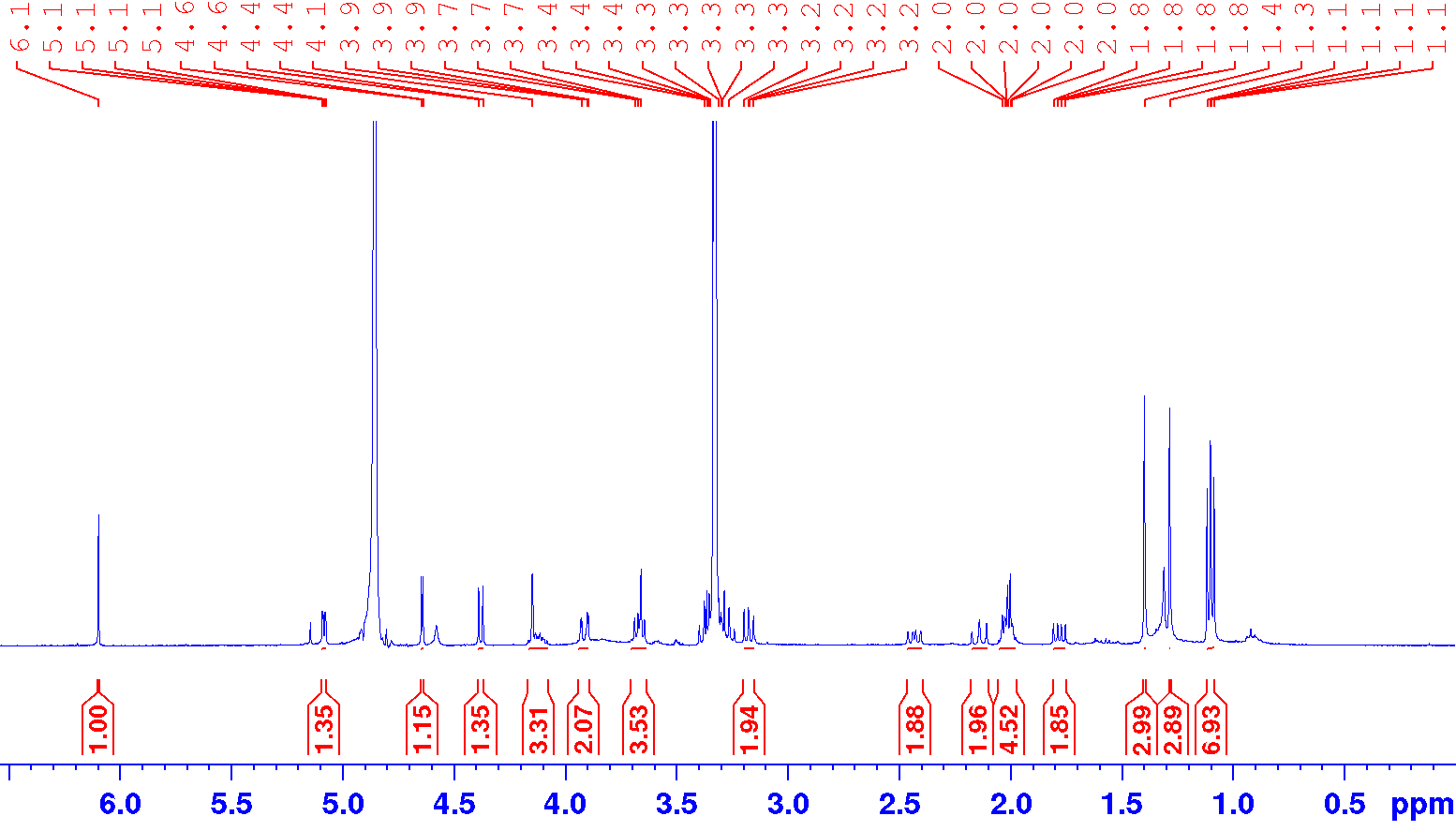


**Figure S13.** ^1^H NMR spectra of **1** (top) and **2** (bottom) measured in Met-*d_4_*, 400 MHz


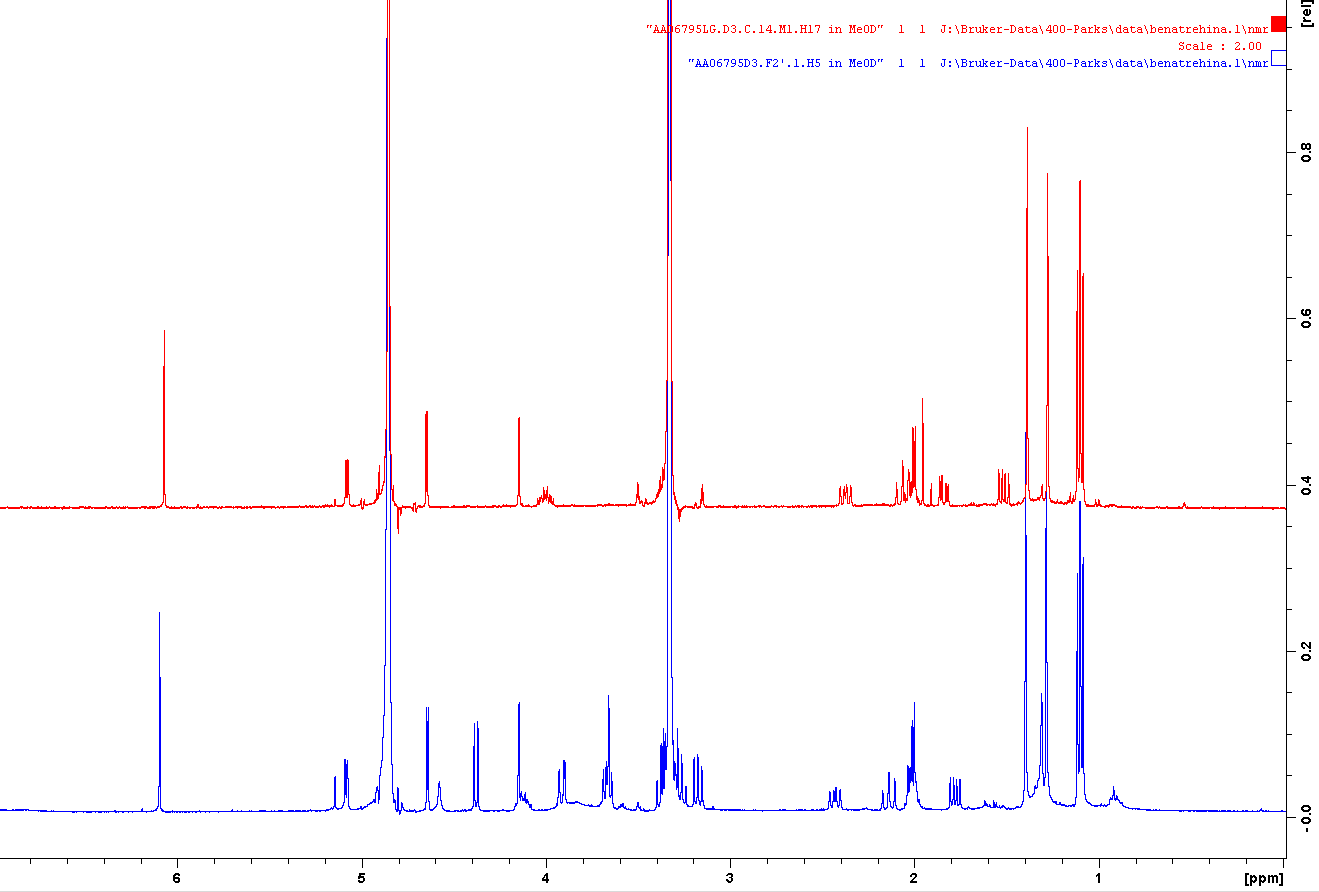

**1** R: *β*-OH

**2** R: *β*-*O*-glc

**Figure S14.** ^13^C NMR spectra of **2** measured in pyr-*d_5_*, 100 MHz


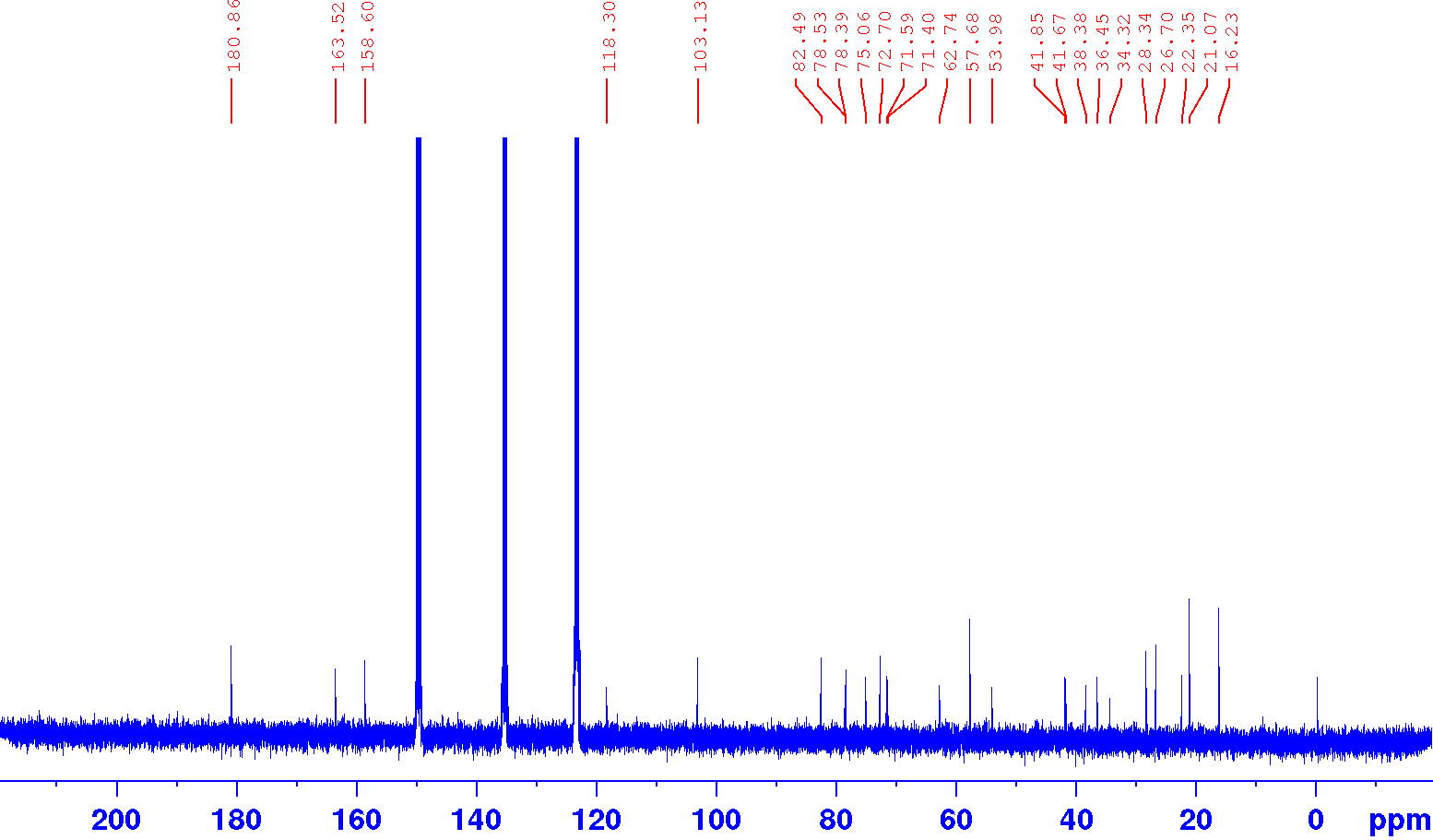

**Figure S15.** HSQC spectrum of **2** measured in pyr-*d_5_*


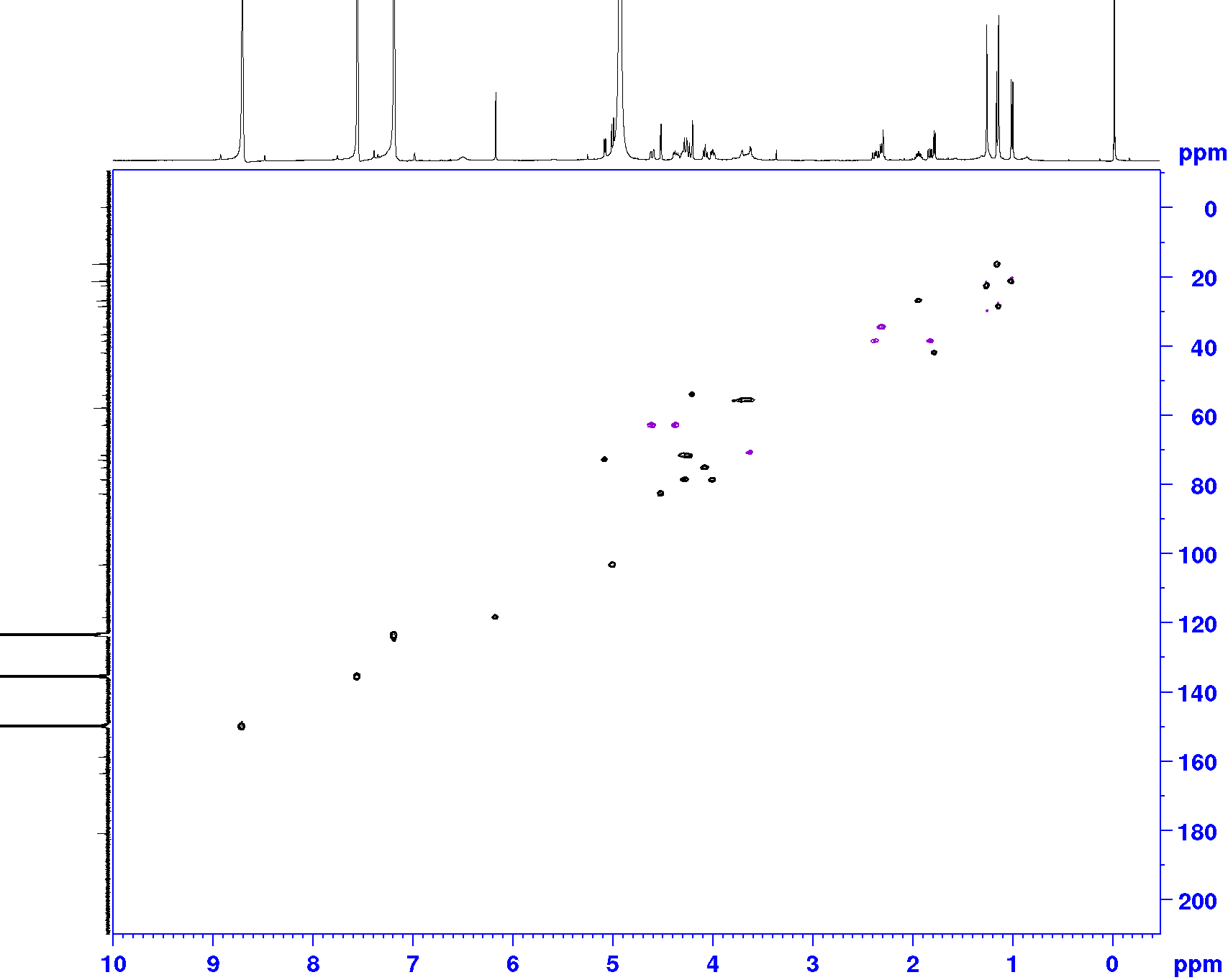

**Figure S16.** HMBC spectrum of **2** measured in pyr-*d_5_*


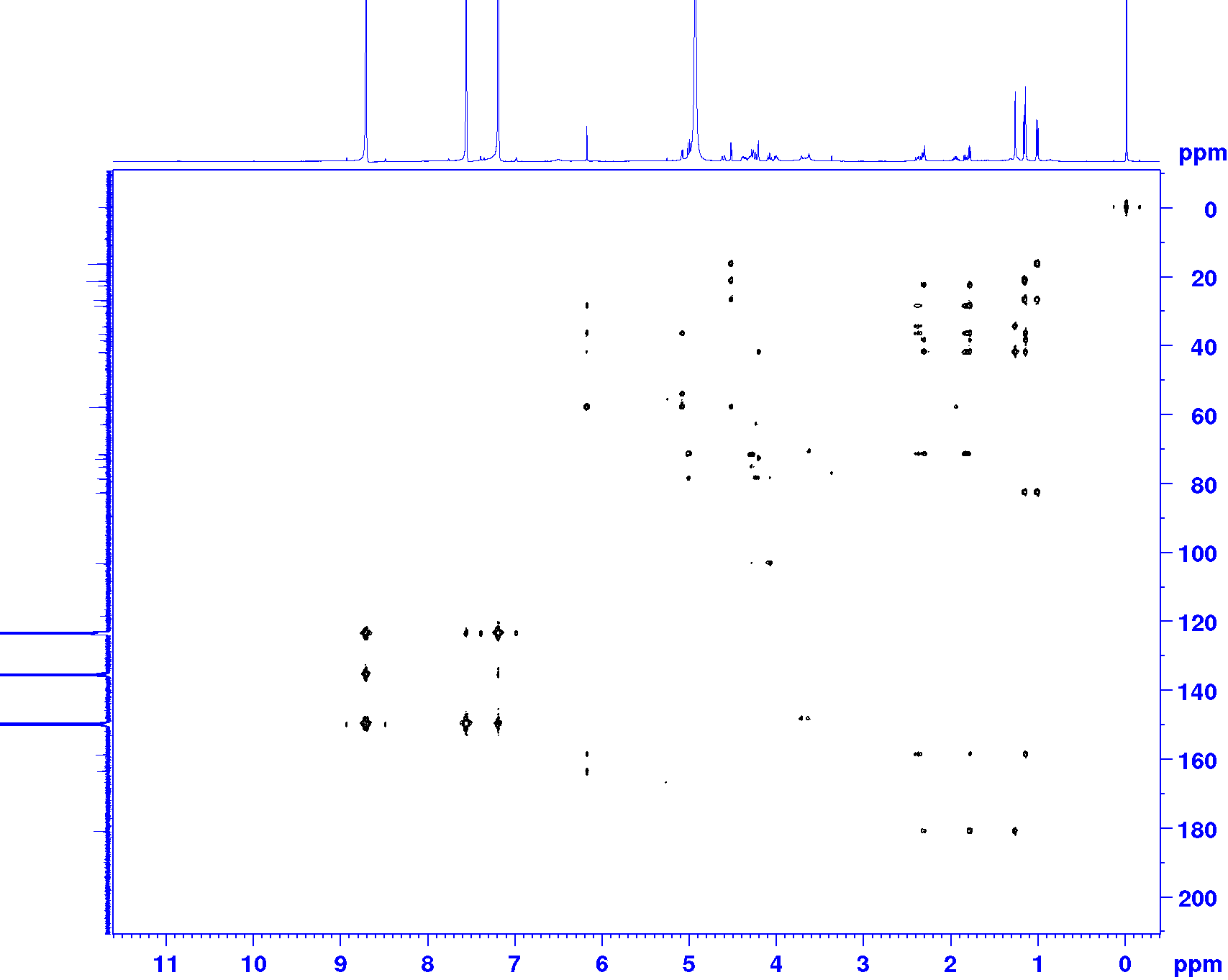

Supplement: Supplementary file 1 — Supplementary material 1 (DOCX 1089 kb) [file 13659_2019_198_MOESM1_ESM.docx]
